# When Should the Ovaries Be Removed?

**Published:** 1887-12

**Authors:** William Goodell


### WHEN SHOULD THE OVARIES BE REMOVED?

By WILLIAM GOODELL, M. D.,

This patient has been in the medical department, in the ophthalmological department, in the department for affections of the skin on account of a bullous eruption, which makes its appearance at the menstural periods, and she has finally been returned to the gynæological department. The question is, is she malingering, or is there a foundation for her complaints? We had in the hospital about a year ago, a patient who went the rounds of all the departments without being much improved. Finally she returned in a deplorable condition and insisted upon having the ovaries removed, and stated if I would not perform the operation, she would have it performed by some one else. When the ovaries were removed, there were distinct evidences of disease, which I had not been able to make out, and the patient has been much better ever since.

Our present patient is twenty-eight years of age, married and has one child. The menses are irregular, sometimes not appearing for three or four months. On vaginal examination, I find the uterus retroverted and very tender. To the right of the uterus there is a tender body. I introduce the sound with care and find that the uterus is to a certain extent mobile, and after it is raised up, I can find that the left ovary is enlarged and roughened with exudates. With the information which I have obtained from her physician, and that which I have been able to gain by my own examination, I am sure that she has had a salpingitis and perio-varitis, and that she should have the ovaries removed. I am, however, very conservative in regard to this operation, and I do not perform it in one-fourth of the cases sent to me to have their ovaries removed. Further, I make it a rule to explain fully all the dangers of operation. For instance: A few years ago, I received a telegram stating that two ladies had started from Colorado to see me, and that one of them required removing of the ovaries. The patient arrived, and I learned that it was a case of epilepsy, the attacks being more frequent at the menstrual periods, but also occurring at other times. She had been told by her physician that if the overies were removed, she would recover. She actually thought that the operation was a very simple one, and that it could be performed off-hand, and that she could return home in a few days. When I told her that there was risk in the operation, and that she would have to stay with me for four weeks, the complexion of things was altered, and that afternoon they started back for Colorado. Many patients seem to think that the operation is one of slight importance, and I think that physicians are, to a certain extent, to blame for this. In any operation in which there is danger to life, this should be stated before the operation is performed. There may be cases in which it would not be prudent that the patient should be informed of the full danger of the operation, but some member of the family should be. Again, the operation may be absolutely necessary, and yet the patient may not be in a mental condition to decide the question.

I often find it a perplexing question to decide when the ovaries should be removed and when they should not be. Fortunately, most of the cases sent to me are those of functional trouble and are relieved by the "rest treatment." In these cases there is this left ovarian pain and often prolapse of the left ovary or of both ovaries, yet they will recover under treatment. A very few need the removal of the ovaries. This operation does not unsex them, although it does prevent them becoming mothers. In suitable cases, I employ the "rest treatment" with massage and electricity, and the majority of cases in which the patients are young girls, recover. Even in gonorrhœeal cases, with organic lesions, you may get the patient comparatively well by these means. However, where there is positive and persistent organic disease, you will usually be forced to operate.

Sometime ago I was requested to go to Kansas to remove the ovaries. I was so busy at the time I could not leave. Arrangements were then made to bring the patient half-way and I was to meet her. I found a patient with excessive ovarian pain, with terrible dysmenorrhœa and reduced to skin and bone. She had been bed-ridden for two years, and was unable to sleep or eat. I suggested to her physician who accompanied her, that it would be best in the first place to relieve the stenosis and cure the dysmenorrhœa. The left ovary was also lower than normal. There was, however, no history of pelvic peritonitis, or of organic disease of the ovary. The constant congestion was sufficient to account for displacement of the ovary. The decision was that she was placed under my care, and she came to this city. I dilated the cervix and put her on the "rest treatment" with massage and electricity. At the next period, the dysmenorrhœa was excessive, for the uterus had not recovered from the bruises produced by the operation. The next period was better, and after this she continued to improve, and before long began to walk. Last week I received from her husband a most grateful letter. In these cases the symptoms point so distinctly to the ovaries as the seat of the disease, that unless you are on your guard you will be deceived. Any woman subjected to great mental trouble, is liable to manifest symptoms referable to the ovaries. A young girl, for six months, nursed her father, who was suffering with cancer of the lip. After his death, she broke down and presented very exciting symptoms of uterine and ovarian trouble. She came to me to have a good diagnosis made by her physician reversed. He had told her that the whole trouble was due to nervous prostration, and I fully confirmed his opinion. Sometimes I err on the conservative side, yet all these cases are, in a measure, improved by the "rest treatment," and, if necessary, the operation can be performed subsequently.

If a patient is put at absolute rest, without exercise, harm will be produced after the first week or two. To compensate for this, exercise is given by massage. If a skilled rubber can be obtained, any strong woman can improve the circulation by pinching, stroking, kneading and rubbing the skin and muscles, especially if she is given some instruction by the physician. At first the movements should be confined to stroking and to soothing movements, but after a time more energetic manipulations must be resorted to. Accompanying this, the use of electricity is serviceable. There is no question but that the action of electricity extends deeper than that of massage. It is probable, although not yet positively proven, that the strength of the nervous system may be toned up by the application of electricity. We do not yet know what nerve fluid is, but in its behavior it is more analogous to electricity than to any other agent with which we are acquainted. In addition to this, it is well to give tonics. It is often difficult to determine when iron should be given. Sometimes the tongue continues to be furred and is only cleaned by the use of iron in large doses. I usually begin with the tincture of iron with dilute phosphorus acid, and to this may be added strychnine in doses of from one-fortieth to one-twentieth of a grain. If this acts well, it may be followed by Bland's pills, the effect being carefully watched. In this way many cases will recover.—*College aud Clinical Record.*

